# Seihai-to (TJ-90)-Induced Activation of Airway Ciliary Beatings of Mice: Ca^2+^ Modulation of cAMP-Stimulated Ciliary Beatings via PDE1

**DOI:** 10.3390/ijms19030658

**Published:** 2018-02-26

**Authors:** Haruka Kogiso, Yukiko Ikeuchi, Masako Sumiya, Shigekuni Hosogi, Saori Tanaka, Chikao Shimamoto, Toshio Inui, Yoshinori Marunaka, Takashi Nakahari

**Affiliations:** 1 Department of Molecular Cell Physiology, Graduate School of Medical Science, Kyoto Prefectural University of Medicine, Kyoto 602-8566, Japan; hkogiso@koto.kpu-m.ac.jp (H.K.); ikeuchi@koto.kpu-m.ac.jp (Y.I.); fw12b22@stu.kpu-m.ac.jp (M.S.); hosogi@koto.kpu-m.ac.jp (S.H.); marunaka@koto.kpu-m.ac.jp (Y.M.); 2 Laboratory of Pharmacotherapy, Osaka University of Pharmaceutical Sciences, Takatsuki 569-1096 Japan; stanaka@gly.oups.ac.jp (S.T.); shimamoto@gly.oups.ac.jp (C.S.); 3 Saisei Mirai Clinics, Moriguchi 570-0012, Japan; toshio.inui1003@gmail.com; 4 Department of Bio-Ionomics, Graduate School of Medical Science, Kyoto Prefectural University of Medicine, Kyoto 602-8566, Japan; 5 Japan Institute for Food Education and Health, St. Agnes’ University, Kyoto 602-8013, Japan

**Keywords:** airway, cilia, PDE1, dynein, cAMP, Ca^2+^

## Abstract

Sei-hai-to (TJ-90, Qing Fei Tang), a Chinese traditional medicine, increases ciliary beat frequency (CBF) and ciliary bend angle (CBA) mediated via cAMP (3′,5′-cyclic adenosine monophosphate) accumulation modulated by Ca^2+^-activated phosphodiesterase 1 (PDE1A). A high concentration of TJ-90 (≥40 μg/mL) induced two types of CBF increases, a transient increase (an initial increase, followed by a decrease) and a sustained increase without any decline, while it only sustained the CBA increase. Upon inhibiting increases in intracellular Ca^2+^ concentration ([Ca^2+^]_i_) by 10 μM BAPTA-AM (Ca^2+^-chelator, 1,2-Bis(2-aminophenoxy)ethane-*N*,*N*,*N*′,*N*′-tetraacetic acid tetrakis(acetoxymethyl ester) or Ca^2+^/calmodulin-dependent PDE1 by 8MmIBMX (a selective PDE1 inhibitor), TJ-90 (400 μg/mL) induced only the sustained CBF increase without any transient CBF increase. The two types of the CBF increase (the transient increase and the sustained increase) induced by TJ-90 (≥40 μg/mL) were mimicked by the stimulation with both procaterol (100 pM) and ionomycin (500 nM). Thus, TJ-90 stimulates small increases in the intracellular cAMP concentration ([cAMP]_i_) and [Ca^2+^]_i_ in airway ciliary cells of mice. These small increases in [cAMP]_i_ and [Ca^2+^]_i_ cause inducing a transient CBF increase or a sustained CBF increase in an airway ciliary cells, depending on the dominant signal, Ca^2+^-signal, or cAMP-signal.

## 1. Introduction

The mucociliary clearance, which is conducted by the beating cilia cooperating with the surface mucous layer, is a host defense mechanism of the lungs. The surface mucous layer entraps inhaled small particles and the beating cilia transport the mucous layer with the small particles to the oropharynx. Thus, the beating cilia are the key apparatus to conduct the mucociliary clearance [1,2,3]. The ciliary beating is activated by an accumulation of cAMP and an increase in intracellular Ca^2+^ concentration ([Ca^2+^]_i_) [2,3,4]. Many drugs activating the ciliary beatings, such as β_2_-agonists and PDE inhibitors, are used to improve respiratory problems.

Qing Fei Tan (Seihai-to (TJ-90) in Japanese), which is a Chinese traditional medical mixture (16 herbs), is used for improving chdronic cough, abundant sticky mucous phlegm, and sore throat, and is also used for the treatment of the patients having bronchitis, bronchial asthma, and pneumonia [5,6]. TJ-90 has a variety of biological activities, such as xanthine oxidase activity [6,7,8]. The previous study revealed that TJ-90 increases ciliary beat frequency (CBF) and Cl^−^ secretion in the rabbit trachea [9]. However, it remains uncertain how TJ-90 increases CBF and Cl^−^ secretion in the airway epithelia.

The previous studies in *Chlamydomonas* revealed that cilia have two functionally distinct molecular motors: inner dynein arms (IDAs) regulating waveform, including ciliary bend angle (CBA), and outer dynein arms (ODAs), regulating ciliary beat frequency (CBF) [10,11]. Activities of the ciliary beating are assessed by two parameters, CBF and CBA [12,13,14,15,16,17,18], and an increase in CBF or CBA has already been shown to activate the ciliary transport of the lung airway surface [12]. We found that TJ-90 increased both CBF and CBA in mice lung airway ciliary cells, and, moreover, that TJ-90 at a high concentration (400 μg/mL) induced a transient CBF increase (an initial increase followed by a decrease), although it induced a sustained CBA increase.

Previous reports demonstrated that the Ca^2+^-dependent phosphodiesterase 1A (PDE1A) controls cAMP accumulation in the CBF-regulating metabolon of airway cilia [15,16,17,18]. If TJ-90 stimulates an [Ca^2+^]_i_ increase and a cAMP accumulation, then the [Ca^2+^]_i_ increase would evoke two CBF responses: (1) an increase in CBF via the direct Ca^2+^ action; and (2) a decrease in CBF via PDE1A activation (the indirect Ca^2+^ action) [18]. The indirect action (PDE1 activation), which stimulates cAMP degradation, inhibits the cAMP-regulated CBF increase or decreases the rate of cAMP-regulated CBF increase [15,16,17,18]. The goal of this study is to clarify the effects of TJ-90 on the CBF and CBA increases, especially the transient CBF increase.

## 2. Results

### 2.1. Effects of TJ-90 on CBA and CBF

Supplementary Material S1 and S2 show isolated airway ciliary cells of mouse recorded by a video-microscope equipped with a high speed camera. The video images recorded by a high speed camera (500 fps) clearly show the fine movement of beating cilia. Stimulation with TL-90 (400 μg/mL) increased CBF and CBA (Supplementary Material S1 and S2). The image analysis program enabled us to measure the frequency (CBF) and the angle (CBA) of beating cilia.

#### 2.1.1. Two Types of CBF Increase Stimulated by TJ-90

**Figure 1:**
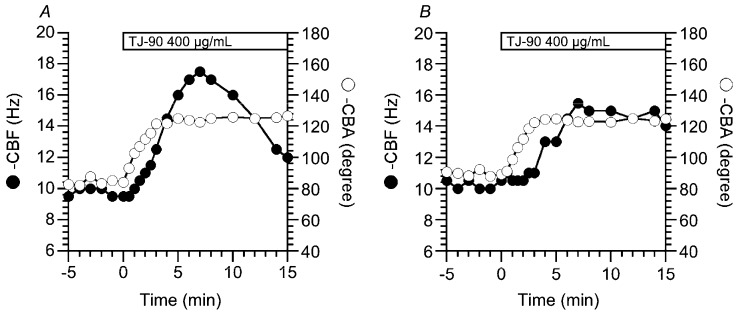
Effects of Sei-hai-to (TJ-90) (400 μg/mL) on ciliary beat frequency (CBF) and ciliary bend angle (CBA) in airway ciliary cells. TJ-90 induced two types of CBF increase, an initial increase followed by a decrease (transient increase) and an initial increase followed by a sustained increase (sustained increase), although it only sustained the CBA increase. (**A**) A typical case showing the transient CBF increase and the sustained CBA increase; (**B**) a typical case showing the sustained CBF increase and CBA increase.

TJ-90 at 400 μg/mL induced two types of CBF increase, a transient increase (an initial increase followed by a decrease) (Figure 1A), and a sustained increase without any decline (Figure 1B), although it only induced a sustained CBA increase (Figure 1A,B). Figure 1A shows a typical case showing the transient CBF increase. In this case, stimulation with TJ-90 (400 μg/mL) initially increased CBF from 10 to 18 Hz, and then decreased to 12 Hz, while it increased CBA from 82° to 126° and sustained, without any decrease (Figure 1A). Figure 1B also shows a typical case showing the sustained CBF increase. In this case, stimulation with TJ-90 (400 μg/mL) initially increased CBF from 10 to 15 Hz and then sustained without any decrease, and also increased CBA from 86° to 123°, and then sustained. In both cases, the CBF increases were slower in the time course than the CBA increases. Previous studies demonstrated that an [Ca^2+^]_i_ increase reduces the rate of CBF increase, not the rate of CBA increase, stimulated by cAMP accumulation. The slower CBF increase than CBA increase is caused by an activation of PDE1A existing in the metabolon regulating CBF [17,18]. Moreover, TJ-90 at 400 μg/mL evoked cell shrinkage (V/V_0_ 15 min from the start of TJ-90 stimulation = 0.83 ± 0.03 (*n* = 3)). The cell shrinkage is evoked by an [Ca^2+^]_i_ increase in many cell types [19]. These observations suggest that TJ-90 stimulates cAMP accumulation and [Ca^2+^]_i_ increase.

**Figure 2:**
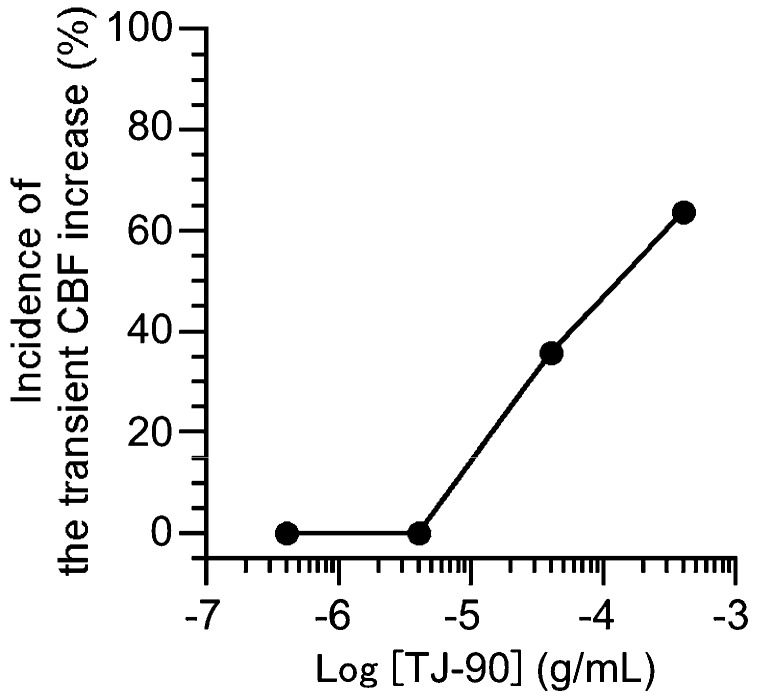
Concentration-effects of TJ-90 on the incidence rate of transient CBF increase (%). Increment of TJ-90 concentration elevated the incidence rate of transient CBF increase.

We examined the concentration effects of TJ-90 on the incidence rate of transient CBF increase. The incidences of the transient CBF increase were 14/22 experiments at 400 μg/mL TJ-90, 5/14 experiments at 40 μg/mL TJ-90, 0/8 experiments at 4 μg/mL TJ-90, and 0/11 experiments at 0.4 μg/mL TJ-90. The incidence rate (%) of transient CBF increase was plotted against the TJ-90 concentration (Figure 2). The elevation of TJ-90 concentration increased the incidence rate of transient CBF increase.

#### 2.1.2. Concentration Effects of TJ-90 on the CBF Increase and CBA Increase

**Figure 3:**
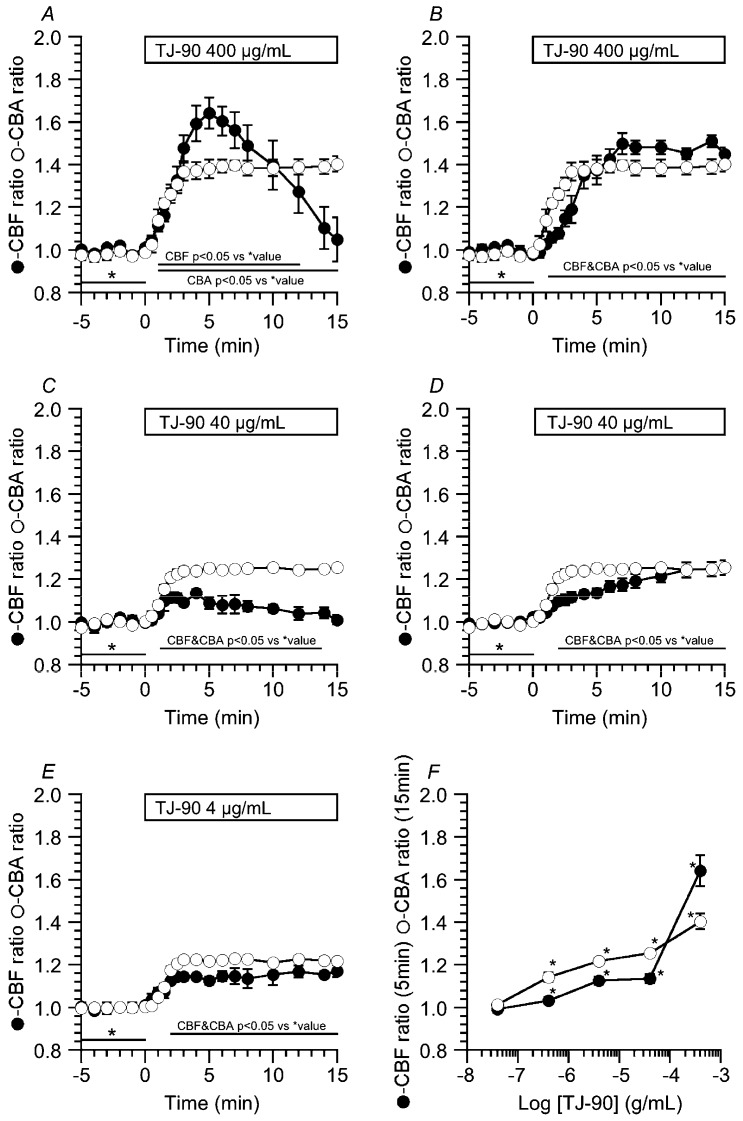
Concentration-effects of TJ-90 on the CBF increase and CBA increase. The CBF ratio (=CBF_t_/CBF_0_) and the CBA ratio (=CBF_t_/CBF_0_) were calculated to normalize changes in CBA and CBF. TJ-90 at concentrations higher than 40 μg/mL induced two types of CBF increase (a transient CBF increase and a sustained CBF increase), although it only sustained the CBA increase. (**A**,**B**) TJ-90 (400 μg/mL) induced a transient CBF increase (an initial increase followed by a decrease) (**A**) and a sustained CBF increase (**B**) and it sustained the CBA increase; (**C**,**D**) TJ-90 (40 μg/mL) still induced two types of CBF increase and also sustained the CBA increase. Changes in CBA in panel B or D were re-plotted from panel A or C, respectively; (**E**) TJ-90 (4 μg/mL) sustained the CBF increase and the CBA increase. * shows control values (**A**–**E**) (**F**) Concentration response study of TJ-90. The CBF ratio 5 min from the start of TJ-90 stimulation and CBA ratio 15 min from the start of TJ-90 stimulation were plotted. The CBF ratio and the CBA ratio were obtained from 4 to 13 cells at each TJ-90 concentration. * significantly different from the values of CBA ratio and CBF ratio before TJ-90 stimulation (*p* < 0.05).

The CBF ratio (=CBF_t_/CBF_0_) or the CBA ratio (=CBA_t_/CBA_0_), which is the normalized CBF or CBA to the control value, was calculated to compare changes in CBF and CBA among experiments. Figure 3A,B show changes in the CBF ratio and the CBA ratio stimulated by TJ-90 (400 μg/mL). TJ-90 at 400 μg/mL induced a transient CBF increase (Figure 3A) and a sustained CBF increase (Figure 3B), although it only sustained the CBA increase. In the transient CBF increase, the CBF ratios 5 min and 15 min from the start of TJ-90 stimulation were, respectively, 1.64 ± 0.07 and 1.05 ± 0.10 (*n* = 8), and the CBA ratio 15 min from the start of TJ-90 stimulation was 1.40 ± 0.02 (*n* = 5) (Figure 3A). In the sustained CBF increase, the CBF ratio 15 min from the start of TJ-90 stimulation was 1.43 ± 0.03 (*n* = 5) (Figure 3B). TJ-90 at 40 μg/mL induced a transient CBF increase (Figure 3C) and a gradual CBF increase (Figure 3D), while it only sustained the CBA increase. In the transient CBF increase (Figure 3C), the CBF ratios 4 min and 15 min from the start of TJ-90 stimulation were 1.13 ± 0.02 and 1.01 ± 0.02 (*n* = 5), and the CBA ratio 15 min from the start of TJ-90 stimulation was 1.26 ± 0.01 (*n* = 4). In the gradual CBF increase (Figure 3D), the CBF ratio 15 min from the start of TJ-90 stimulation was 1.25 ± 0.03 (*n* = 9). TJ-90 at 4 μg/mL only sustained the CBF increase and the CBA increase (Figure 3E). The CBF ratio and CBA ratio 15 min from the start of TJ-90 stimulation were 1.17 ± 0.03 (*n* = 8) and 1.22 ± 0.01 (*n* = 5). Figure 3F shows the concentration effects of TJ-90 on CBF and CBA. The CBF ratios 5 min from the start of TJ-90 stimulation and the CBA ratios 15 min from the start of TJ-90 stimulation were plotted against TJ-90 concentrations. The CBF ratio and CBA ratio at each TJ-90 concentration were obtained from 4 to 13 cells. TJ-90 increased CBF and CBA in a concentration dependent manner.

#### 2.1.3. Effects of a PKA Inhibitor (PKI-A) on CBA Increase and CBF Increase Stimulated by TJ-90

**Figure 4:**
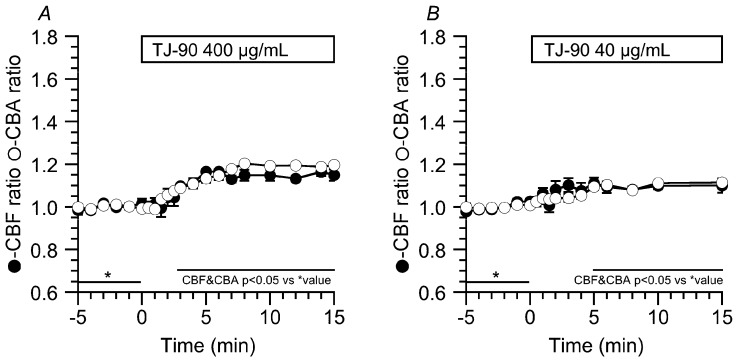
Inhibition of the CBF increase and the CBA increase stimulated by TJ-90 by PKI-A (2 μM, a PKA inhibitor). Prior addition of PKI-A inhibited the CBF increase and the CBA increase stimulated by TJ-90 (400 and 40 μg/mL). However, in the presence of PKI-A, TJ-90 did not induce any CBF decrease. (**A**) TJ-90 (400 μg/mL, *n* = 4); and, (**B**) TJ-90 (40 μg/mL, *n* = 4). * shows control values.

To inhibit PKA, the cells were treated with 2 μM PKI-A for 10 min prior to the TJ-90 stimulation. The addition of PKI-A alone did not induce any change in CBF or CBA. The prior addition of PKI-A inhibited increases in CBF and CBA stimulated by TJ-90 (400 μg/mL), and the CBF ratio and CBA ratio 15 min from the start of TJ-90 stimulation were 1.15 ± 0.03 (*n* = 4) and 1.19 ± 0.00 (*n* = 4), respectively (Figure 4A). The prior addition of PKI-A also inhibited increases in CBF and CBA stimulated by TJ-90 (40 μg/mL), and the CBF ratio and CBA ratio 15 min from the start of TJ-90 stimulation were 1.10 ± 0.03 (*n* = 4) and 1.11 ± 0.01 (*n* = 4), respectively (Figure 4B). Thus, the prior PKI-A treatment decreased the extent of CBA increase and CBF increase stimulated by TJ-90 (400 or 40 μg/mL) by 60–70%. However, in the presence of PKI-A, stimulation with TJ-90 (400 or 40 μg/mL) never decreased CBF (Figure 4). This suggests that the transient CBF increase, especially the CBF decrease following to the initial increase, stimulated by TJ-90 would be a PKA-dependent process, suggesting a decrease in cAMP concentration induces the CBF decrease.

### 2.2. cAMP Contents and [Ca^2+^]_i_

**Figure 5:**
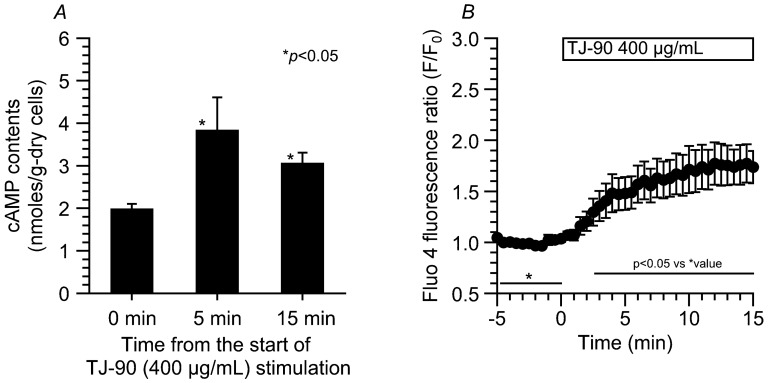
Effects of TJ-90 on cAMP contents and [Ca^2+^]_i_. (**A**) The contents of cAMP in isolated lung cells stimulated with TJ-90. In each experiment, 3 mice were used. TJ-90 (400 μg/mL) increased cAMP contents in isolated lung cells. * significantly different from the control value (*p* < 0.05); (**B**) Changes in [Ca^2+^]_i_ (*n* = 4). TJ-90 (400 μg/mL) gradually increased [Ca^2+^]_i_. * shows control values.

Hochu-ekki-to (TJ-41), a mixture of 10 herbs, five of which are mixed in the TJ-90, has been shown to increase CBF via cAMP accumulation in airway epithelial cells [20]. We hypothesized that TJ-90 may also stimulate cAMP accumulation. We measured the cAMP contents in the cells isolated from lungs during TJ-90 stimulation. The method measuring cAMP contents has already been described in the previous reports [17,18]. TJ-90 (400 μg/mL) increased cAMP contents in the cells isolated from lungs (Figure 5A). The contents of cAMP 5 min from the start of TJ-90 stimulation appear to be larger than those 15 min from the start of the stimulation, although both values are not significantly different.

We also measured [Ca^2+^]_i_ in airway ciliary cells during TJ-90 stimulation. Changes in [Ca^2+^]_i_ were measured by fluo 4, and expressed by the fluorescence ratio (F/F_0_) [21]. The TJ-90 (400 μg/mL) gradually increased [Ca^2+^]_i_ (Figure 5B). The F/F_0_s stimulated by TJ-90 did not exceed 2.0. Thus, the increases in [Ca^2+^]_i_ stimulated by TJ-90 were much less than those stimulated by 1 μM ionomycin (IM), which alone increased F/F_0_ to 3.1 ± 0.14 (*n* = 3).

### 2.3. Effects of BAPTA-AM and 8MmIBMX on the TJ-90-Induced Transient CBF Increase

**Figure 6:**
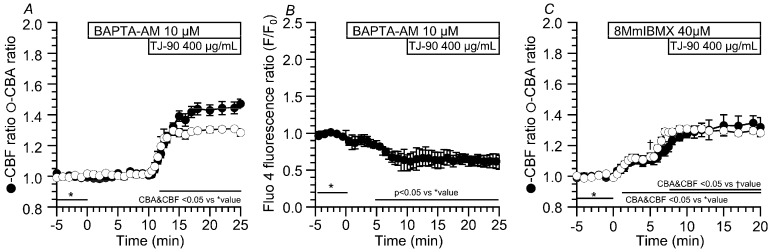
Effects of BAPTA-AM (10 μM) and 8MmIBMX on CBA and CBF. (**A**) The addition of BAPTA-AM did not change CBA and CBF. The further addition of TJ-90 (400 μg/mL) induced a rapid increase followed by a sustained increase in CBA and CBF without any decrease in CBF; (**B**) Changes in [Ca^2+^]_i_. The addition of BAPTA-AM decreased [Ca^2+^]_i_ (*n* = 4). The further addition of TJ-90 did not induce any change in [Ca^2+^]_i_; (**C**) Effects of 8MmIBMX (40 μM, a selective PDE1 inhibitor) on CBA and CBF. The addition of 8MmIBMX increased CBA and CBF. The further addition of TJ-90 (400 μg/mL) induced a sustained CBF increase without any decrease, and also induced a sustained CBA increase. The time courses of CBF and CBA increase were similar. * shows control values (**A**–**C**) and † shows the values just before the addition of TJ-90 (**C**).

An [Ca^2+^]_i_ increase appears to decrease CBF by activating PDE1A in airway ciliary cells [17,18], upon stimulation with TJ-90 (≥40 μg/mL). Cells were pretreated with 10 μM BAPTA-AM (an intracellular Ca^2+^ chelator), which inhibits any increases in [Ca^2+^]_i_ [18,21]. In the previous study, BAPTA-AM at 5 μM has already been shown to induce no change in CBF and CBA in airway ciliary cells [18]. In this study, the addition of 10 μM BAPTA-AM did not induce any change in CBF and CBA. The further addition of TJ-90 (400 μg/mL) induced sustained the CBF increase without any decrease (Figure 6A). The CBF ratio and CBA ratio 15 min from the start of TJ-90 stimulation were 1.47 ± 0.03 (*n* = 10) and 1.28 ± 0.03 (*n* = 4), respectively. We also measured [Ca^2+^] is using fluo 4-loaded airway ciliary cells. The experimental protocol was the same as Figure 6A. The addition of BAPTA-AM (10 μM) decreased [Ca^2+^]_i_, and the further addition of TJ-90 (400 μg/mL) did not induce any increase in [Ca^2+^]_i_ (Figure 6B). No transient CBF increase was noted in the presence of 10 μM BAPTA-AM, upon stimulation with TJ-90 (400 μg/mL). Thus, the prior treatment of BAPTA-AM (10 μM), a decrease in [Ca^2+^]_i_, induced a sustained CBF increase without any CBF decrease, no transient CBF increase, during stimulation with TJ-90 (400 μg/mL). This suggests that an [Ca^2+^]_i_ increase causes the following CBF decrease in the transient CBF increase stimulated by TJ-90. The previous studies showed that the Ca^2+^-dependent PDE1A regulates CBF by controlling cAMP concentration in the CBF-regulating metabolon of the airway cilia during procaterol stimulation and that PDE1A activated by an increased [Ca^2+^]_i_ decreases CBF [17,18].

We examined the effects of a PDE1 inhibitor (40 μM, 8-methoxymethyl-3-isobutyl-1-methylxanthine (8MmIBMX)) on the CBF increase and the CBA increase stimulated by TJ-90 (Figure 6C). The addition of 40 μM 8MmIBMX alone increased CBA and CBF, as previously reported [17,18]. The CBF ratio and CBA ratio 5 min after the addition of 8MmIBMX were 1.11 ± 0.03 (*n* = 6) and 1.12 ± 0.02 (*n* = 4). The further TJ-90 (400 μg/mL) stimulation sustained the CBF increase and the CBA increase. The CBF and CBA reached plateaus within 3 min from the start of TJ-90 stimulation, and the time courses of CBF increase was similar to that of CBA increase, in the presence of 8MmIBMX (Figure 6C). The CBF ratio and CBA ratio 15 min from the start of TJ-90 stimulation were 1.11 ± 0.03 (*n* = 6) and 1.12 ± 0.02 (*n* = 4) (Figure 6C). Previous studies demonstrated that, in the presence of 8MmIBMX, procaterol (a β_2_-agonist) increased CBF and CBA in similar time courses [17,18]. Moreover, in the presence of 40 μM 8MmIBMX, no transient CBF increase was noted upon stimulation with TJ-90 (400 μg/mL). Thus, 8MmIBMX (40 μM, an inhibitor of Ca^2+^-dependent PDE1), induced the sustained CBF increase without any CBF, no transient CBF increase, during stimulation with TJ-90 (400 μg/mL). These results suggest that an [Ca^2+^]_i_ increase that is stimulated by TJ-90 causes to decrease CBF by an activation of PDE1A (cAMP degradation in the CBF-regulating metabolon).

### 2.4. Effects of Procaterol and Ionomycin on Increases in CBF and CBA

**Figure 7:**
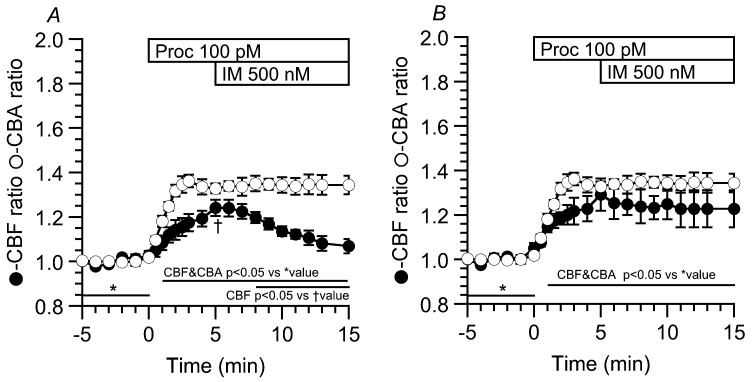
Effects of ionomycin (500 nM, IM) on CBF increase and CBA increase stimulated by procaterol (100 pM). The stimulation with 100 pM procaterol increased and sustained CBF and CBA by ~35%. The further addition of 500 nM IM decreased CBF (7/10 cells) or did not change CBF (3/10 cells), although it only sustained the CBA increase. (**A**) Cells were first stimulated with 100 pM procaterol for 5 min, and then, further with 500 nM IM. The addition of 500 nM IM decreased the CBF ratio gradually (*n* = 4), but not CBA (*n* = 3). The incidence was 7/10 cells; (**B**) The further addition of 500 nM IM did not change the CBF ratio (*n* = 3), and also the CBA ratio (*n* = 3). The incidence was 3/10 cells. * shows control values and † shows the values just before the addition of IM (A).

To examine the effects of an [Ca^2+^]_i_ increase on the cAMP-stimulated CBF increase, airway ciliary cells were first stimulated with procaterol (an β_2_-agonist) and then with ionomycin (IM). First, cells were with 1 nM procaterol and then with 1 μM IM. Procaterol (1 nM) increased CBF by 80%, but the further addition of 1 μM IM only decreased it by 10%, as previously reported [18]. We also examined the effects of 10 μM IM on increases in CBF and CBA stimulated by 100 pM procaterol. The addition of 10 μM IM increased CBF and CBA unlike the case of 1 μM IM [18]. Thus, IM at a high concentration, such as 10 μM, induced no CBF decrease (CBF/CBA ratio 10 min after the IM stimulation = 1.42 ± 0.10/1.39 ± 0.04, *n* = 3). In the next experiment, we used 100 pM procaterol and 500 nM IM. Airway ciliary cells were first stimulated with 100 pM procaterol and then with 500 nM IM. The stimulation with 100 pM procaterol increased CBF and then, the further stimulation with 500 nM IM gradually decreased CBF in 7/10 cells (Figure 7A). However, in 3/10 cells, the further stimulation with IM did not change CBF (Figure 7B). The CBA increases stimulated by procaterol (100 pM) were not affected by the further IM stimulation (500 nM). Thus, two types of the CBF increases induced by TJ-90 (≥40 μM), a transient CBF increase (an initial increase, followed by a decrease) and a sustained CBF increase, were mimicked by 100 pM procaterol and 500 nM IM. These results indicate that small increases in cAMP and [Ca^2+^]_i_ induce both the transient CBF increase and the sustained CBF increase, depending on the balance of cAMP-signal and Ca^2+^-signal.

## 3. Discussion

The present study demonstrates that TJ-90 increases CBF and CBA mediated via cAMP accumulation and [Ca^2+^]_i_ increase in airway ciliary cells of mice. An accumulation of cAMP plays an essential role in the CBF increases (a transient CBF increase and a sustained CBF increase) and the CBA increase during stimulation with TJ-90, because both CBF and CBA increases that are stimulated by TJ-90 are inhibited by a PKA inhibitor. An [Ca^2+^]_i_ increase stimulated by TJ-90 modulates the cAMP accumulation.

The previous studies exhibited that an accumulation of cAMP sustains the CBF increase in airway ciliary cells [12,17,18,22]. However, TJ-90 at high concentrations (≥40 μg/mL) induced two types of CBF increase, the transient CBF increase and the sustained CBF increase. TJ-90 (1 mg/mL) has been shown to induce a transient CBF increase in rabbit tracheal ciliary cells in primary culture [9]. The transient CBF increase, especially a decrease following to an initial increase, stimulated by TJ-90 was caused by an increase in [Ca^2+^]_i_. This suggests that an [Ca^2+^]_i_ increase suppresses cAMP accumulation during TJ-90 stimulation.

Previous studies demonstrated that the cAMP concentration in the metabolon regulating CBF is controlled by Ca^2+^/calmodulin-dependent PDE1A in the airway cilia [17,18]. Ionomycin (1 μM) inhibited the CBF increase stimulated by procaterol (200 pM) in airway ciliary beating, indicating that an [Ca^2+^]_i_ increase inhibits cAMP accumulation via PDE1A activation [18]. This inhibition of CBF increase is explained as follows; an [Ca^2+^]_i_ increase induces an activation of PDE1A in the cilium, which decreases cAMP concentration in the metabolon regulating CBF, leading to no CBF increase. TJ-90 at a high concentration also appears to produce the similar experimental condition. TJ-90 at high concentrations initially stimulates cAMP accumulation, leading to increase CBF. The following [Ca^2+^]_i_ increase induces an activation of PDE1A, which decreases cAMP concentration in the metabolon regulating CBF in the cilium, leading to the CBF decrease. Thus, during TJ-90 stimulation, PDE1A activated by an [Ca^2+^]_i_ increase caused the CBF increase to be transient.

On the other hand, TJ-90 also induced not only the transient CBF increase, but also the sustained CBF increase. In the case of sustained CBF increase, cAMP degradation by PDE1A would be negligibly small and an increased cAMP concentration would be maintained in the metabolon regulating CBF. In this study, stimulation with both 100 pM procaterol and 500 nM IM also evoked the two types of CBF increases similarly to stimulation with TJ-90 (400 μg/mL). However, high concentrations of procaterol (≥1 nM) and IM (≥1 μM) failed to induce two types of CBF increases [18]. Thus, for producing two types of CBF increases, small increases in intracellular concentration of cAMP and [Ca^2+^]_i_ are required. Under these limited conditions, the balance between Ca^2+^-signal or cAMP-signal would be altered in experimental conditions or cellular conditions and determines the CBF response, transient or sustained. A small shift to the Ca^2+^ signal side in the balance between cAMP signal and Ca^2+^ signal would produce the transient CBF increase, and to the contrary, a small shift to the cAMP signal side would produce the sustained CBF increase.

On the other hand, TJ-90 induced only the sustained CBA increase. In the cilium, PDE1A exists in the metabolon regulating CBF, not in the metabolon regulating CBA [17]. Therefore, a small [Ca^2+^]_i_ increase does not decrease cAMP concentration in the CBA-regulating metabolon although it decreases cAMP concentration in the CBF-regulating metabolon. Thus, a small [Ca^2+^]_i_ increase stimulated by TJ-90 does not affect CBA increased by cAMP accumulation. Similar CBA increases in various [Ca^2+^] have been shown in the previous reports [17,18].

TJ-90 is a mixture of 16 herbs containing a lot of substances, such as biological active polyphenolic compounds [23,24], and has a variety of pharmacological actions, such as xanthine oxidase activities [5], inhibition of leukotriene C4 synthesis [25], IL 4 and IL6 production on human monocyte [8], and interferon induction via glycyrrhizin [7]. The TJ-90 actions are unlikely to be explained by the sum of each TJ-90 ingredient action. Each compound contained in TJ-90 appears to have the interactions in vitro and in vivo, which may induce variety of cell responses, such as [Ca^2+^]_i_ mobilization and cAMP accumulation in airway ciliary cells. To confirm this point, further studies are required.

TJ-90 is widely used for preventing chronic respiratory problems, such as recurrent aspiration pneumonia [5,6,26] and allergic rhinitis [27]. These reports suggest that TJ-90 has not only acute actions, but also chronic actions. There are many ingredients in TJ-90, some of which, such as glycyrrhizin inhibiting 11-β-hydroxysteroid dehydrogenase, appear to have genomic actions [7]. These actions may play a role for preventing recurrent aspiration pneumonia and allergic rhinitis. Further studies are required.

## 4. Materials and Methods

### 4.1. Ethical Approval

The procedures and protocols for the experiments were approved by the Committee for Animal Research of Kyoto Prefectural University of Medicine (No. 26-263, 12th April 2017). The animals were cared for and the experiments were carried out according to the guidelines of this committee. Female mice (C57BL/6J, 5 weeks of age) were purchased from Shimizu Experimental Animals (Kyoto, Japan) and fed standard pellet food and water ad libitum. The mice were used for experiments until 10 weeks of age. The mice were first anesthetized by inhalational isoflurane (3%), and were then further anesthetized and heparinized by an intraperitoneal injection (ip) of pentobarbital sodium (40 mg/kg) plus heparin (1000 units/kg) for 15 min. Finally, the mice were sacrificed by a high-dose pentobarbital sodium (100 mg/kg, ip).

### 4.2. Solution and Chemicals

The solution was composed of 121 mM NaCl, 4.5 mM KCl, 25 mM NaHCO_3_, 1 mM MgCl_2_, 1.5 mM CaCl_2_, 5 mM NaHEPES, 5 mM HEPES, and 5 mM glucose. The pHs of solutions were adjusted to 7.4 by adding 1 M HCl or 1M NaOH. All solutions were aerated with a gas mixture (95% O_2_ and 5% CO_2_) at 37 °C. TJ-90 was a generous gift from Tsumura & Co. (Tokyo, Japan). The procaterol was a generous gift from Otsuka Pharmaceutical Co., Ltd. (Tokyo, Japan). Heparin, elastase, ionomycin, and bovine serum albumin (BSA) were purchased from Wako Pure Chemical Industries, Ltd. (Osaka, Japan), BAPTA-AM and fluo 4-AM were from Dojindo Laboratories (Kumamoto, Japan), and PKI 14-22 amide (PKI-A) was from Tocris (Bristol, UK), 8-methoxymethyl-3-isobutyl-1-methylxanthine (8MmIBMX, a selective inhibitor of PDE1) was from Calbiochem (Darmstadt, Germany), and DNase I and dimethyl sulfoxide (DMSO) were from Sigma Chemical Co. (St Louis, MO, USA). The reagents were dissolved in DMSO or water as appropriate. The final DMSO concentration did not exceed 0.1%, and DMSO at this concentration has no effect on cellular events, including CBA and CBF [12,13,14,15,16,17,18,22,28]. TJ-90 was added in the water heated at 85 °C (50 mg/mL) for 15 min and then the mixture was centrifuged for 10 min (3000 rpm). The supernatant was filtered and then stocked at −20 °C. TJ-90, and the reagents were prepared to their final concentrations immediately before the experiments.

### 4.3. Cell Preparations

Female mice (C57BL/6J, 5 weeks of age) were purchased from Shimizu Experimental Animals (Kyoto, Japan) and fed with standard pellet food and water ad libitum. Lung epithelial cells, including airway ciliary cells, were isolated from the lungs, as previously described [12,17,18,22,28]. After the sacrifice, the lungs were cleared of blood by perfusion via the pulmonary artery using a nominally Ca^2+^-free solution containing heparin (20 units/mL), and the lungs, together with the trachea and heart, were removed from the mouse en bloc. The procedure for cell isolation from the lungs has already been described in details elsewhere [12,17,18,22]. Briefly, the lung cavity was washed with a nominally Ca^2+^-free solution and then with the control solution. After the washing, the lung cavity was digested with the control solution containing elastase (0.2 mg/mL) and DNase I (0.02 mg/mL) for 40 min at 37 °C. After this treatment, lungs were minced using fine forceps in the control solution containing DNase I (0.02 mg/mL) and BSA (5%), and the minced tissue was filtered through a nylon mesh (a sieve having 300 μm openings). The isolated cells were washed three times with centrifugation (160× *g* for 5 min) and were then re-suspended in the control solution at 4 °C. The cells were used for experiments within 5 h after the isolation.

### 4.4. CBA and CBF Measurements

The cells were set on a coverslip precoated with Cell-Tak (Becton Dickinson Labware, Bedford, MA, USA) in a microperfusion chamber (20 μL) mounted on an inverted light microscope (T-2000, NIKON, Tokyo, Japan) connected to a high-speed camera (IDP-Express R2000, Photron Ltd., Tokyo, Japan). The experiments were carried out at 37 °C, because the CBF is temperature-dependent [29]. The chamber was perfused with the control solution aerated with a gas mixture (95% O_2_ and 5% CO_2_ at 37 °C) at a constant rate (300 μL/min). The ciliary cells were distinguished from other lung epithelial cells by their beating cilia. Ciliary cells accounted for around 10–20% of isolated lung cells. For the CBA and CBF measurements, video images were recorded for 2 s at 500 fps. Video images of airway ciliary cells are shown in the supplemental files (Supplementary Material S1 and S2). The previous reports have already described the method in details to measure CBA and CBF [12,17,18]. Before the start of experiments, the cells were perfused with the control solution for 5 min and then with test solutions containing various drugs. After the experiments, CBA and CBF were measured using an image analysis program (DippMotion 2D, DITECT, Tokyo, Japan) [12]. The CBA and CBF ratios (CBA_t_/CBA_0_ and CBF_t_/CBF_0_: normalized CBA and CBF) were calculated to make comparisons across the experiments. Five CBFs or CBAs measured every 1 min during 5 min control perfusion were averaged and the averaged value was used as CBF_0_ or CBA_0_. The subscripts “0” and “t” indicate, respectively, just before and the time from the start of experiments. Each experiment was carried out using 6–10 coverslips with cells obtained from 3 to 5 animals. For each coverslip, we selected a visual field with 1–2 cells or a cell-block and measured their CBAs or CBFs. The ratios of CBA and CBF were calculated by averaging values obtained from 3 to 6 cells (“*n*” shows the number of cells).

### 4.5. Measurement of cAMP Contents

Isolated lung cells were incubated in test solutions for 5 min or 15 min at 37 °C. The test solution contains DMSO (vehicle control) or 400 μg/mL TJ-90. After the experiments, the cells were immediately frozen by the liquid nitrogen and stored at −80 °C until the cAMP measurements. The cAMP contents in the cells were measured using a cAMP EIA (Cayman Chemical, Ann Arbor, MI, USA) and the weight of dry cells were measured. The cAMP contents were expressed as nmoles/gram-dry cells.

### 4.6. Measurement of the Cell Volume

For the cell volume measurements, the outline of a ciliary cell was traced on a video image, and the area (A) was measured. The index of cell volume (*V*_t_/*V*_0_ = (*A*_t_/*A*_0_)^1.5^) was calculated [22]. The indices of cell volume measured every 1 min during the control perfusion (5 min) were averaged and the averaged value was used as V_0_. The subscript “0” or “t” indicates the time before or after the start of experiments, respectively. Each experiment was carried out using 4–6 cover slips obtained from two to three animals. “*n*” shows the number of cells.

### 4.7. Measurement of [Ca^2+^]_i_

Intracellular Ca^2+^ concentration ([Ca^2+^]_i_) was measured using fluo 4 fluorescence. Isolated ciliary cells were incubated with 5 μM fluo4-AM (fluo4-acetoxymethyl ester) for 30 min at 37 °C. The fluo 4 fluorescence was measured using a confocal laser scanning microscope (model LSM 510META, Carl Zeiss, Jena, Germany). The fluo 4 was excited at 488 nm and the emission was 510 nm. The fluo 4 fluorescence ratio (*F*_t_/*F*_0_) was calculated. The subscripts “0” or “t” indicate the time just before and from the start of experiments, respectively.

### 4.8. Administration of TJ-90 for Examining the Chronic Effects on CBA and CBF

TJ-90 was added in the water heated at 85 °C (72 mg/mL) for 15 min and then the mixture was centrifuged for 10 min (3000 rpm). The supernatant was stocked at −20 °C. The stock was prepared to its final concentration (7.2 mg/mL) just before the administration. The TJ-90 solution (7.2 mg/mL) was administered orally by free drinking. Mice, body weights of which were approximately 30 g, were fed with a standard pellet food. The TJ-90 solution (200 mL) was set in the cage, in which 5 mice were kept. The TJ-90 solution was replaced with the new one every 1 week. The consumed volume of TJ-90 solution was measured every one week. Five mice consumed 150 mL of the TJ-90 solution a week. The results indicate that a mouse takes TJ-90 approximately 1 g/kg/day. The control group mice were kept with a standard pellet food and water containing no TJ-90.

### 4.9. Statistical Analysis

Data are expressed as the means ± standard error (SEM). Statistical significance between the means was assessed by analysis of variance (ANOVA), Student’s paired or unpaired *t*-test, as appropriate. Differences were considered significant at *p* < 0.05. The statistical significances are shown in the figures.

## Supplementary Materials

Supplementary materials can be found at http://www.mdpi.com/1422-0067/19/3/658/s1.
