# Supplementary material for: Seihai-to (TJ-90)-Induced Activation of Airway Ciliary Beatings of Mice: Ca2+ Modulation of cAMP-Stimulated Ciliary Beatings via PDE1
Source: Int J Mol Sci. 2018 Feb 26;19(3):658. doi: 10.3390/ijms19030658 (PMC5877519; doi:10.3390/ijms19030658)
Supplement: Supplementary file 1 [file ijms-19-00658-s001.zip › ijms-261470-supplementary/Supplemental_files/supplemetary captions.docx]

**Supplementary Materials:**

S1: Isolated airway ciliary cells: ciliary beating of airway ciliary cells before the TJ-90 stimulation

S2: Isolated airway ciliary cells: ciliary beating of airway ciliary cells 3 min after the TJ90 (400 µg/mL) stimulation
